# Descriptive study: Feasibility of integrating hypertension screening into HIV assisted partner notification services model in Kenya

**DOI:** 10.1097/MD.0000000000033067

**Published:** 2023-02-22

**Authors:** Jerusha N. Mogaka, Harrison Lagat, George Otieno, Paul Macharia, Beatrice Wamuti, Sarah Masyuko, Monisha Sharma, Edward Kariithi, Carey Farquhar, Tecla M. Temu

**Affiliations:** a School of Nursing, University of Washington, Seattle, WA; b PATH, Kisumu, Kenya; c Department of Global Health, University of Washington, Seattle, WA; d Harvard School of Public Health, Harvard University, Boston; e Ministry of Health-National AIDS and STI Control Program, Nairobi, Kenya; f Department of Epidemiology and Medicine, University of Washington, Seattle, WA; g Institute of Tropical Diseases, University of Nairobi, Nairobi, Kenya.

**Keywords:** assisted partner notification service, care, HIV, hypertension, integration, Kenya, men, sub-Saharan Africa, treatment

## Abstract

Prevalence of hypertension (HTN) and human immunodeficiency virus (HIV) are high among men while screening rates are low. Assisted partner notification service is a strategy recommended by the World Health Organization that aims to increase HIV testing and treatment uptake and may present an opportunity to offer integrated HIV/HTN screening and treatment services. In this prospective cohort study, we assessed the feasibility of integrating HTN screening for male sexual partners of females newly tested HIV-positive in 10 health facilities in Kenya. Participants were notified of the exposure and offered HIV testing and HTN screening; if they accepted and tested positive for either HTN, HIV, or both, they were referred for care. HTN was defined as systolic blood pressure ≥ 140 mm Hg, diastolic blood pressure ≥ 90, or the use of antihypertensive medication. Among 1313 male partners traced, 99% accepted HIV testing and HTN screening. Overall, 4% were found to have HTN, 29% were in the pre-HTN stage, and 9% were HIV-positive. Only 75% had previously been screened for HTN compared to 95% who had previously tested for HIV. A majority preferred non-facility-based screening. The participants who refused HTN screening noted time constraints as a significant hindrance. HIV and HTN screening uptake was high in this hard-to-reach population of men aged 25 to 50. Although HTN rates were low, an integrated approach provided an opportunity to detect those with pre-HTN and intervene early. Strategic integration of HTN services within assisted partners services may promote and normalize testing by offering inclusive and accessible services to men.

## 1. Introduction

Hypertension (HTN) is a major modifiable risk factor for cardiovascular diseases. It contributes to 45% of all heart disease deaths and 51% of stroke related deaths globally.^[1–3]^ HTN prevalence has been increasing steadily in sub-Saharan Africa (SSA), with a 41% rise seen between 2000 to 2010 which is anticipated to increase by 60% by 2030.^[4,5]^ Significant gaps have been documented in the detection, awareness, treatment, and control of HTN in Kenya, particularly among men. Over 23.8% of Kenyan adults have HTN; however, only 1 in 5 are aware of their status and <3% of those aware have their blood pressure (BP) under control.^[6]^

In Kenya, men are less likely to seek screening for HTN compared to women (36% vs 63%) who have routine interactions with service providers during pregnancy, the postnatal period, and when seeking reproductive health services.^[7–10]^ In light of the gaps in screening for HTN and human immunodeficiency virus (HIV) among men, the Kenya Ministry of Health and the World Health Organization issued a call to develop novel strategies to integrate HTN screening into existing platforms such as HIV care and treatment services.^[11]^ Despite these recommendations, HTN screening is rarely integrated into HIV testing services.^[12,13]^

Assisted partner notification services (aPNS) is a global public health strategy recommended by the World Health Organization that involves tracing and offering HIV testing services to sexual partners.^[14]^ aPNS aims to increase HIV testing and treatment uptake and presents a unique opportunity to offer HTN screening alongside HIV/acquired immunodeficiency syndrome services.^[15]^ This pilot study sought to evaluate the feasibility of integrating HTN screening services into the aPNS by targeting male partners of females testing HIV-positive in western Kenya.

## 2. Methods

### 2.1. Project description and study setting

This prospective cohort study was nested within the ongoing aPNS scale-up project. The project was conducted by preventing and treating HIV-Kenya and the University of Washington in collaboration with the Ministry of Health Kenya; in 30 health facilities in Kisumu and Homa Bay counties in Kenya.^[16]^ The objective of this aPNS study is to evaluate the effectiveness and acceptability of integrating aPNS into existing HIV testing services. A detailed description of the project procedures has been published.^[16]^ In summary, the aPNS project enrolled females ≥ 15 years of age and newly diagnosed with HIV (female index client). Male sexual partners of these female index clients were contacted by HIV testing service (HTS) providers and invited to screen for HIV. The male partners who test positive for HIV received a 1-year post enrollment follow-up to assess linkage to care and treatment.^[16]^ Simultaneously, in 10 of the 30 healthcare facilities, HTN screening was offered to all male partners ≥ 18 years who provided verbal consent. These health facilities were randomly chosen as a representative sample of the high volume versus low volume sites and urban versus rural areas in Kisumu and Homa Bay Counties.

### 2.2. Study procedures and data collection

HTS providers anonymously notified male partners of the HIV exposure by phone or in person and offered HTN screening and HIV testing at the nearest hospital, home, or convenient venue. Informed consent was obtained before data collection. A questionnaire was used to collect demographic characteristics, HTN screening history, and additional details on those who declined HTN screening. Using a digital automatic BP monitor, 2 BP measurements were obtained on the right arm after the subject was seated in a resting position for at least 5 minutes. The lower reading of the 2 was recorded and used for analysis. A rapid HIV test was used to confirm HIV infection. Participants testing HIV positive and/or with elevated BP readings were referred to link to care in an HIV clinic or a regular outpatient clinic. Enrollment and engagement in care for HTN were confirmed during follow-up phone calls made by HTS providers 6 weeks post enrollment.

### 2.3. Outcome measures

Feasibility outcome measures included the proportion of male partners in the aPNS study that; Were offered combined HTN/HIV screening; Accepted HIV/HTN screening, and; Screened positive for HTN, HIV, or both. The proportion of individuals with HTN who were linked to care and initiated effective antihypertensive therapy was included. HTN was defined as a mean systolic BP of ≥ 140 mm Hg or a diastolic BP of ≥ 90 mm Hg, or self-report of previous HTN diagnosis by a health care provider and currently taking antihypertensive drugs within the last 2 weeks as per the Kenyan guidelines.^[17]^ Pre HTN was defined as systolic BP of 130 to 139 mm Hg or diastolic BP of 80 to 89 mm Hg.

### 2.4. Data analyses

Sociodemographic characteristics were described using medians or percentages. Categorical variables were described using proportions, while continuous variables used medians and interquartile ranges. Proportions were used to quantify the feasibility of integrating HTN screening with and without HIV testing. All analyses were conducted using STATA (College Station, TX).

### 2.5. Ethical approval

Ethical approval was obtained from the University of Washington institutional review board and the Kenyatta National Hospital ethics review committee. Verbal informed consent was obtained from the male partners before BP screening.

## 3. Results

### 3.1. Participant’s characteristics

**Figure 1.**
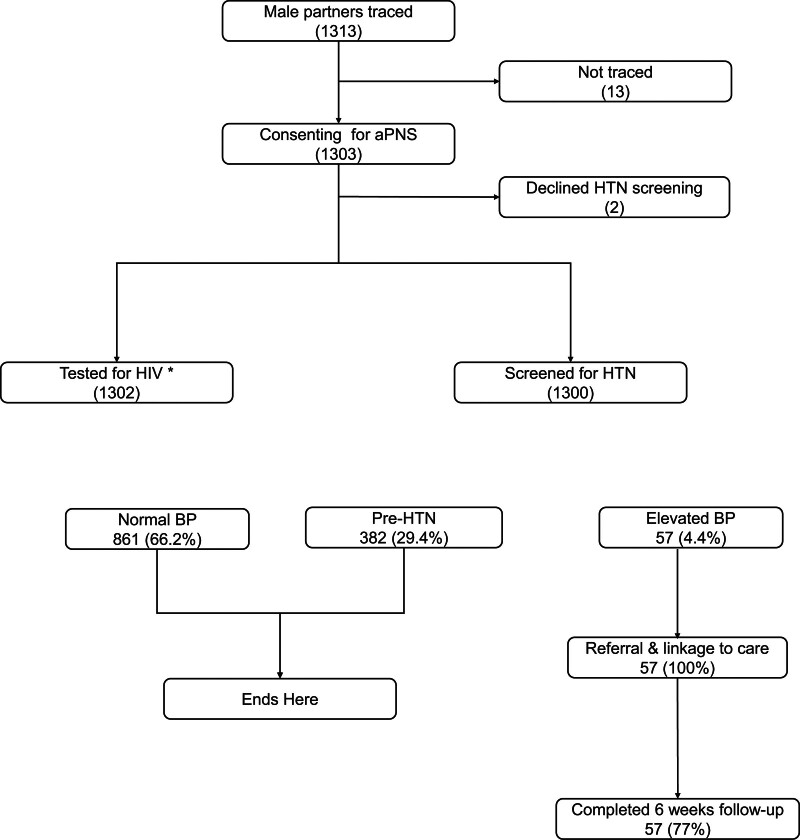
Study flow chart highlighting screening, enrollment, and follow-up activities after partner tracing in the aPNS. *One participant declined HIV testing. HTN-BP ≥ 140/≥90 mm Hg. Pre-HTN-systolic BP of 130-139/80-89 mm Hg. aPNS = assisted partner notification services, BP = blood pressure, HIV = human immunodeficiency virus, HTN = hypertension.

Between September 2020 and April 2021, 1313 male partners were traced and 1303 were enrolled (Fig. 1). Table 1 represents the sociodemographic characteristics of male partners. The median age was 38, and most (77%) were married. The median number of male partners for each female index was 3.

**Table 1:** Sociodemographic characteristics of male participants.

Characteristics	Male partners N = 1303	
Age (yr), median (IQR)	38	(32,42)
County		
Homa Bay	832	(64)
Kisumu	471	(36)
Marital status		
Single	152	(12)
Married monogamous/cohabiting	1005	(77)
Married polygamous	87	(7)
Divorced/separated/widowed	59	(4)
Highest Education completed		
Never attended school	5	(0)
Primary school	364	(28)
Secondary school	730	(56)
postsecondary school	204	(16)
Occupation		
Employed/self-employed	1190	(91)
Unemployed	66	(5)
Student	47	(4)
Monthly average income ($)		
0–100	596	(46)
>100	707	(54)
Screening strategy		
Clinic	392	(30)
Home	793	(61)
Workplace	109	(8)
Others	6	(1)
Ever screened for HTN		
Yes	975	(75)
No	328	(25)
Ever screened for HIV		
Yes	1233	(95)
No	70	(5)
HIV status		
Newly tested HIV positive	128	(10)
Negative	738	(57)
Persons living with HIV (known positive)	435	(33)
Unknown	1	(0)

Data are reported as numbers, percentages, or median (interquartile range [IQR]).

ART = antiretroviral therapy, aPNS = assisted partner services, HIV = human immunodeficiency virus, HTN = hypertension.

### 3.2. HTN integration with HIV screening

Of the 1303 male partners who consented to study enrollment, 3 male partners (0.2%) declined HTN screening, citing time constraints and emotional unreadiness as the reason for screening refusal. A quarter of the participants reported having never had their BP checked before; in contrast, only about 5% had never been tested for HIV (Table 1). All participants with a previous HIV diagnosis were on antiretroviral therapy, while only 34% with a known HTN diagnosis were on treatment. A majority (70%) of participants opted for non-facility-based screening.

### 3.3. Elevated blood pressure burden

Of the male partners screened for HTN, 29.4% were in the pre-HTN stage and 4% were hypertensive (Fig. 1). Kisumu County had a significantly higher prevalence of both HTN (5% vs 4% and pre-HTN (39% vs 24%) compared to Homa Bay County, respectively (*P* < .01 for all). At the 6-week follow-up visit, 77% of those with screening readings consistent with HTN confirmed they had their BP rechecked, were linked to care, and had initiated HTN treatment (lifestyle modification and/or antihypertensive medication) at nearby health facilities.

## 4. Discussion

In this study conducted in a real-world setting, we found high HTN and HIV screening uptake among this hard-to-reach population of relatively young male partners. The preferred screening venue was home-based. Compared to HIV, a larger proportion of male partners had never been screened for HTN. Although rates of HTN were generally low, we found a high prevalence of prehypertension. Our study supports the integration of HTN screening into HIV testing through aPNS in Kenya and other low and middle income countries.

The high uptake of both HTN and HIV screening services has previously been reported in studies conducted in SSA countries. Mosha et al^[18]^ reported high uptake of HIV/HTN screening in a community based HIV screening program in Tanzania. Drain et al^[19]^ also demonstrated that it was feasible to integrate HTN screening in HIV voluntary testing sites in South Africa. Our study results prove that integration can be effective. We used an innovative integrated approach that leveraged aPNS to promote HIV/HTN screening among men, a population that has been defined as hard to reach and less likely to present in a hospital setting for routine screening. We found that a larger proportion of male participants had tested for HIV compared to HTN screening. This presents an opportunity to screen for HTN and capture any undiagnosed cases that would otherwise be missed and can potentially increase the number of men who routinely screen for both HTN and HIV.

We found low HTN prevalence which was expected given the younger age of our participants. Of concern was the high prevalence of pre-HTN in this relatively young population of men. This presents an opportunity for earlier intervention through lifestyle modification before progression to HTN. Studies have highlighted that approximately 40% of individuals diagnosed with pre-HTN progress to HTN within 2 years with no intervention.^[20]^ Thus, it is critical to create opportunities for routine screening, capture pre-HTN early, and decrease or stop progression to HTN as the population ages.^[21]^

The current healthcare system offers better opportunities for women to interact with healthcare workers at various stages in life as they are more likely to be screened for diseases such as HTN and HIV during family planning, maternal and childcare service delivery.^[22]^ Current service delivery creates disparities for the men who are less likely to seek healthcare services.^[22]^ Also, hospital staffing shortages lead to long wait times, discouraging otherwise healthy individuals from routine screening. In our study, HTS providers were trained to screen for HTN and refer participants for further management. The utilization of trained nonphysician and community health care providers to screen for HTN and other noncommunicable diseases (NCD) has been successful and feasible in various studies conducted in SSA.^[8,19,23,24]^ This may solve the staffing shortages in healthcare facilities and offer opportunities for alternative screening in the community.

A majority of men preferred home-based screening. Our study allowed flexibility in choosing a screening venue for the participants, unlike similar studies, conducted purely in the community or clinic. Verbal confirmation of linkage of care and BP recheck at 6 weeks in our study was 70% which is relatively high compared to other studies that range from 25% to 49%.^[25–27]^ Studies with longer follow-up periods are needed to assess control of BP after linkage to care.

Our study has several strengths. We targeted a hard-to-reach population of men and screened them for both HTN and HIV, providing evidence that opportunistic screening within the hospital and community setting is feasible and potentially increases routine screening rates among men. We leveraged an existing platform and trained HTS providers to screen and offer referral services, providing evidence for integrating services and task sharing as potential solutions to increasing access to routine NCD screening within the clinical setting. Our study had several limitations. It had a short follow up period with verbal confirmation of linkage. Therefore, we cannot definitively ascertain the linkage to care following an HTN diagnosis.

As the burden of cardiovascular disease increases in SSA, integration of NCD screening services within the community setting and into other platforms such as HTS is necessary to increase the uptake of both HIV and HTN screening among men who are less likely to seek preventive services in a hospital setting yet will engage in care when diagnosed.

## Acknowledgments

We thank Geoffrey Omondi, PATH HTS providers, and the study participants for their contribution.

## Author contributions

**Conceptualization:** Harrison Lagat, George Otieno, Beatrice Wamuti, Sarah Masyuko, Monisha Sharma, Edward Kariithi, Carey Farquhar, Tecla M. Temu.

**Data curation:** Jerusha N. Mogaka, George Otieno, Tecla M. Temu.

**Formal analysis:** Jerusha N. Mogaka, George Otieno.

**Funding acquisition:** Monisha Sharma, Edward Kariithi, Carey Farquhar, Tecla M. Temu.

**Investigation:** Carey Farquhar, Tecla M. Temu.

**Methodology:** Beatrice Wamuti, Sarah Masyuko, Edward Kariithi, Carey Farquhar, Tecla M. Temu.

**Project administration:** Jerusha N. Mogaka, Harrison Lagat, George Otieno, Paul Macharia, Beatrice Wamuti, Sarah Masyuko, Edward Kariithi, Carey Farquhar, Tecla M. Temu.

**Supervision:** Harrison Lagat, George Otieno, Paul Macharia, Beatrice Wamuti, Sarah Masyuko, Monisha Sharma, Carey Farquhar, Tecla M. Temu.

**Validation:** Carey Farquhar.

**Writing – original draft:** Jerusha N. Mogaka.

**Writing – review & editing:** Jerusha N. Mogaka, George Otieno, Paul Macharia, Beatrice Wamuti, Sarah Masyuko, Monisha Sharma, Edward Kariithi, Carey Farquhar, Tecla M. Temu.
